# Move Together Buckinghamshire: protocol for a pragmatic mixed-methods evaluation of a community physical activity programme for older adults with long-term conditions

**DOI:** 10.1136/bmjopen-2026-119845

**Published:** 2026-07-31

**Authors:** Emma Norris, Rebecca Hings, Daniel Bailey, Christina Victor, Nana Anokye, Amrit Banstola, Dynameni Androulaki Korakaki, Alejandra Carrillo, Louise Mansfield

**Affiliations:** 1 Centre for Health and Wellbeing across the Lifecourse, College of Health, Medicine and Life Sciences, Brunel University London, Uxbridge, UK; 2 Department of Health Sciences, Brunel University London, London, UK; 3 Department of Sport, Health and Exercise Sciences, Brunel University London, London, UK; 4 Centre for Physical Activity in Health and Disease, College of Health, Medicine and Life Sciences, Brunel University London, London, UK

**Keywords:** PREVENTIVE MEDICINE, Exercise, Aging

## Abstract

**Abstract:**

**Introduction:**

Physical inactivity is highly prevalent in older adults living in deprived communities, with many living with long-term health conditions. Community-based and tailored physical activity behaviour change programmes are needed to support increased physical activity in this population. This project aims to conduct a pragmatic pre-post mixed-methods evaluation of a community-based physical activity intervention (Move Together Buckinghamshire).

**Methods and analysis:**

Move Together Buckinghamshire is a 3-year community-based physical activity programme for inactive adults aged over 50 years with at least one long-term health condition living in deprived areas of Buckinghamshire, England. Across the 12-week programme, users will receive: (i) one 45-minute in-person one-to-one consultation with a Physical Activity Referral Specialist (PARS), (ii) three check-in contacts with their allocated PARS taking place by email, phone or text and (iii) access to an online platform to identify and book physical activity opportunities in their local area. The programme’s recruitment target is 3000 service users over the 3-year programme. This pragmatic evaluation will assess the first 18 months of programme delivery using a mixed-methods design. A quantitative outcome evaluation will be conducted for secondary data routinely collected within the programme to assess the primary outcome of physical activity (using the single-item physical activity measure), and secondary outcomes of health-related quality of life, loneliness and confidence to engage in physical activity (assessed at baseline, during and at the end of the 12-week programme and at 6-month and 12-month follow-up). A qualitative process evaluation will use focus groups and interviews with service users and interviews with PARS staff delivering the programme and strategic decision-makers. An economic analysis will be undertaken to estimate the resources and costs required to design, set up and deliver the Move Together Buckinghamshire.

**Ethics and dissemination:**

This study has been approved by the College of Health, Medicine and Life Sciences Research Ethics Committee at Brunel University of London. Informed written consent will be obtained from all participants of the evaluation. Results will be disseminated in peer-reviewed publications, scientific conferences, reports, webinars and through local community outlets.

**Registration details:**

This study has been preregistered on Open Science Framework: https://doi.org/10.17605/OSF.IO/EV24C

**STRENGTHS AND LIMITATIONS OF THIS STUDY**

- Move Together Buckinghamshire is a complex intervention combining both in-person and remote components to maximise participant engagement and retention.
- Combines quantitative and qualitative data, providing both measurable outcomes and in-depth insight into service users’ experiences.
- A limitation is that due to financial constraints, it is not possible to include an objective measure of physical activity. This study does not assess physical activity barriers related to common women’s health conditions. This study also recruits service users that are motivated to increase their physical activity which may limit representativeness to the wider population.
- This study does not contain a control group due to its pragmatic design. This study also includes English-speaking participants only, although translated questionnaires were provided.

## Introduction

 Physical activity is estimated to prevent at least 3.9 million premature deaths globally each year.^1^ Just a 5 min/day increase in moderate-to-vigorous physical activity in the least active adults is estimated to prevent 6% of all deaths.^2^ Engagement in physical activity supports the prevention and management of long-term health conditions, such as cardiovascular disease, type 2 diabetes and cancer,^3 4^ as well as anxiety and depression.^5^ Taking up physical activity in mid- and later-life has clear benefits on physical, psychological, cognitive and functional health.^6–8^ WHO and UK Chief Medical Officers guidelines recommend that adults aged 19–64 should undertake at least 150 min of moderate-intensity (such as brisk walking or cycling), or at least 75 min of vigorous-intensity physical activity (such as running), or some equivalent combination of moderate and vigorous-intensity aerobic physical activity, per week, alongside muscle-strengthening activity.^9^ Older adults aged 65 years and over should aim to accumulate 150 min of moderate intensity aerobic activity, building up gradually from current levels, plus weight-bearing activities to maintain muscle and bone health.^10^ However, Sport England’s Active Lives Adults Survey Report 2024 identified that only 63% of adults aged 55–74 and 43% of adults aged over 75 self-reported meeting the guideline for aerobic activity.^11^ Interventions are, therefore, needed to increase physical activity from midlife and into older adulthood to reduce the associated high burden of long-term conditions.^12^

Physical inactivity is particularly prevalent in areas of low-socioeconomic status (SES). Adults living in the most deprived areas are the least active in England, with only 55.5% meeting the guideline for aerobic activity compared with 68.9% in the least deprived areas.^11^ Older adults living in lower SES areas are also likely to perceive their local physical activity facilities to be unsafe and of little value to their age-group.^13^ These socioeconomic inequalities may be compounded by sex-related differences in physical activity, with older women typically reporting lower activity levels and experiencing distinct barriers to participation compared with older men.^14^ Tailored interventions are required to facilitate physical activity in older adults living in deprived areas^15 16^ and are recommended within a lifecourse approach to physical activity promotion.^17^

Use of digital health services delivered through electronic devices, the internet, apps and related digital technology interventions can be an effective way to increase physical activity in older adults.^18^ A recent meta-analysis comprising nine studies (n=2357) showed that older adults using digital health interventions engaged in 53.2 min more physical activity time per week compared with control groups.^19^ However, studies evaluating interventions in older adults living in deprived communities are lacking. Digital health services are widely considered to be accessible, affordable and scalable, supporting older adults with identifying and participating in physical activity opportunities.^20^ A combination of in-person and digital intervention components is recommended to improve uptake and retention in interventions targeted at adults in lower SES^21^: allowing supportive, in-person relationships to be formed while allowing remote, asynchronous access to app-based content. Additionally, behaviour change interventions informed by theory may be particularly effective for increasing physical activity in older adults. The Capability, Opportunity, Motivation-Behaviour (COM-B) model provides a framework for understanding barriers and facilitators to physical activity,^22 23^ while Motivational Interviewing offers a person-centred approach to enhancing motivation and supporting sustained behaviour change.^24^

Move Together Buckinghamshire is a 3-year programme that promotes physical activity to adults aged over 50 years with one or more long-term health condition living in deprived areas of Buckinghamshire. The programme has been funded and developed by Buckinghamshire Council (South-East England, located about 40 miles north of central London) and delivered by their commissioned programme provider Active in the Community (a Community Interest Company (CIC)). The purpose of Move Together Buckinghamshire is to connect older adults with physical activity opportunities in their local area, via one-to-one behaviour change consultations and an online platform. The intervention builds on examples of effective community-based physical activity programmes, such as Move Together Oxfordshire^25^ and Active Herts,^26–29^ which assessed effects on self-reported physical activity, well-being and perceptions of health.

## Study aim

This project aims to conduct a pragmatic mixed-methods evaluation of first 18 months of the Move Together Buckinghamshire physical activity programme delivery. This paper explains the methods that will be used to evaluate the outcomes, processes and costs of delivering the intervention.

## Methods and analysis

### Design

**Figure 1:**
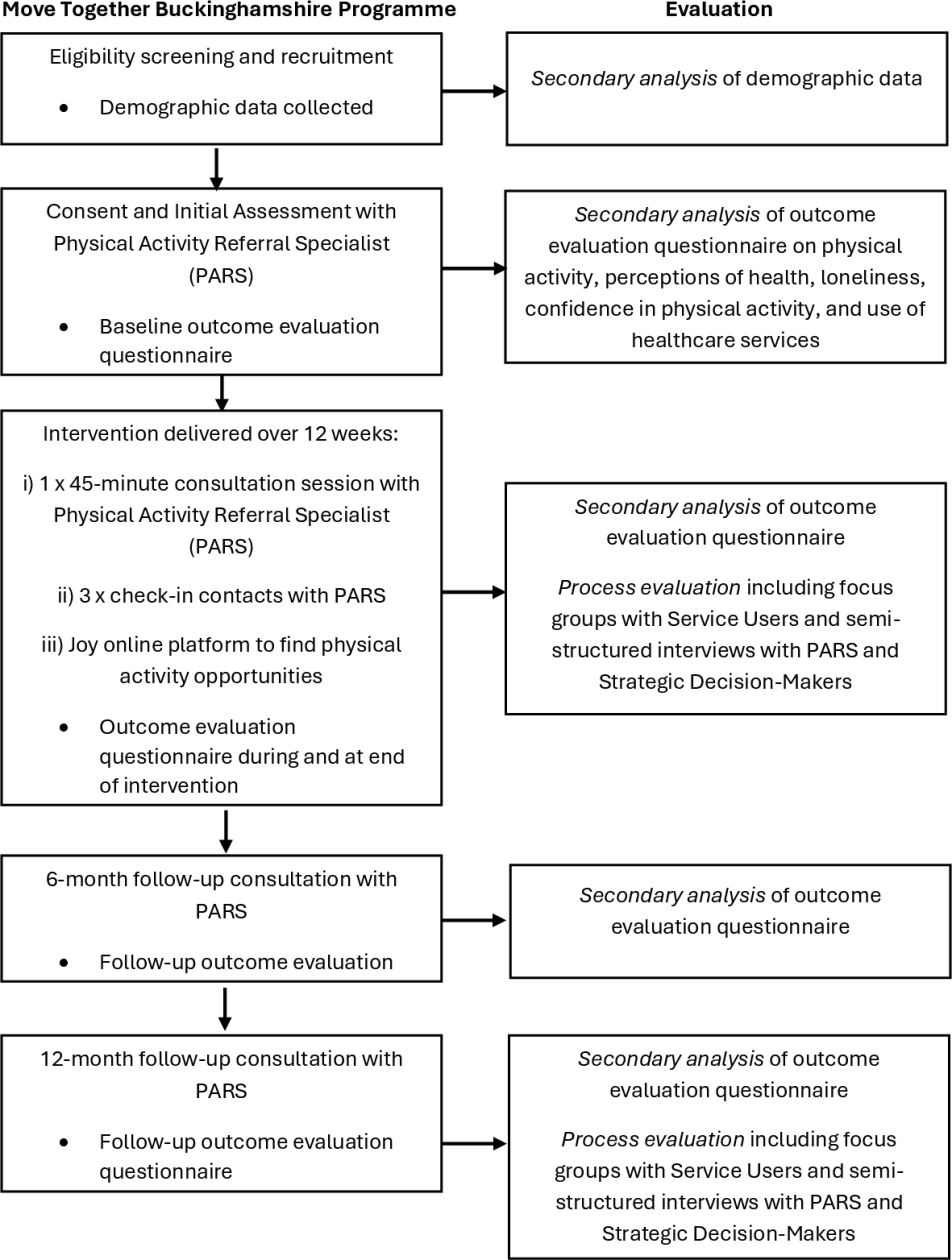
Move Together Buckinghamshire Programme and Evaluation diagram.

The mixed-methods evaluation will include a quantitative outcome evaluation and a qualitative process evaluation. The quantitative study will use a longitudinal observational design, with data collected at baseline, during and at the end of the 12-week programme, and at 6-month and 12-month follow-ups. The qualitative process evaluation will be longitudinal and employ participatory focus groups and in-depth semi-structured interview methods at 3 months and 15 months into the programme (figure 1). A comparator group was not included as no such group was established by Buckinghamshire Council during intervention implementation. Sample size calculations were not performed due to the pragmatic and service evaluation nature of this study.

### Service users

Service users will be recruited into Move Together Buckinghamshire via three pathways: (i) referral from local primary care, such as general practitioners (GPs) and community pharmacists, and secondary care including specialist clinics, (ii) referral through partner organisations of Age UK Buckinghamshire, an independent charity supporting older adults,^30^ and Leap, a social enterprise working to increase physical activity within Buckinghamshire and Milton Keynes,^31^ and (iii) self-referral by signing up through promotions of the programme in the community. The recruitment target set by Buckinghamshire Council is 3000 service users over the 3-year programme.

Service users will be recruited to the programme if they meet the following eligibility criteria: (i) aged over 50 years, (ii) living with a long-term health condition (such as cancer, dementia, diabetes (controlled), hypertension, multiple sclerosis, stroke and musculoskeletal conditions such as osteoporosis, osteoarthritis and fibromyalgia), (iii) currently inactive or fairly active (less than 150 min of moderate physical activity per week) assessed by self-report, (iv) motivated to increase physical activity levels to improve their health and well-being and (v) be a resident in one of the 14 most deprived areas of Buckinghamshire, known as ‘Opportunity Bucks’ wards, and/or registered with one of 17 ‘Deep End’ GP practices^32^ operating in these most deprived areas. Exclusion criteria for participation in Move Together Buckinghamshire include severe asthma and chronic obstructive pulmonary disease, currently accessing cardiac, cancer rehabilitation or pulmonary services, undergoing wider tests regarding a long-term health condition, blood pressure >180 mm Hg (systolic) or >100 mm Hg (diastolic), or resting heart rate >100 bpm. Women with pelvic floor dysfunction, urinary incontinence, prolapse or recent pelvic surgery are not included in the list of eligible long-term health conditions.

The process of screening service users for eligibility will differ depending on the route of referral into the programme. If a health professional (eg, GP) conducts the screening, they are agreeing that the individual’s condition is controlled and they are suitable to take part. When a person self-refers or is referred from a non-clinical setting (eg, an exercise class, a local community centre), the Physical Activity Referral Specialist (PARS) working with the service user will conduct screening during their initial consultation or will redirect the service user to their GP to assess their suitability in borderline cases. PARS will monitor any changes in service users’ long-term health conditions throughout the programme and take appropriate action where such changes affect eligibility to continue participation.

### Move Together Buckinghamshire programme

Move Together Buckinghamshire is being delivered by a programme provider commissioned by Buckinghamshire Council: Active in the Community.^33^ During the 12-week programme, residents meeting the eligibility criteria will receive a one-to-one consultation with a PARS, three check-in contacts with a PARS and access to an online platform to find physical activity opportunities in their local area. Table 1 specifies the behaviour change techniques and modes of delivery used within Move Together Buckinghamshire: characterised by the Behaviour Change Techniques Taxonomy v1 (BCTTv1^34^) and Mode of Delivery Ontology^35^ respectively. No fidelity assessment will be performed within this evaluation.

**Table 1:** Behaviour change techniques and modes of delivery embedded in Move Together Buckinghamshire

Programme component	Behaviour change technique	Content	Mode of delivery
Consultations	Feedback on behaviour (BCT: 2.2) Social support unspecified (BCT: 3.1) Social support emotional (BCT: 3.3) Information about health consequences (BCT: 5.1) Credible source (BCT: 9.1) Verbal persuasion about capability (BCT: 15.1) Focus on past success (BCT: 15.3)	Service users are provided with: one 45-minute in-person one-to-one consultation with a Physical Activity Referral Specialist (PARS).	Face to face (BCIO:011003) Audio call (BCIO:011022) Individual-based (BCIO:011055)
Check-ins	Feedback on behaviour (BCT: 2.2) Social support unspecified (BCT: 3.1) Social support emotional (BCT: 3.3) Credible source (BCT: 9.1) Verbal persuasion about capability (BCT: 15.1) Focus on past success (BCT: 15.3)	Three check-in contacts with their allocated PARS, taking place by email, phone or text.	At-a-distance (BCIO:011004) Individual-based (BCIO:011055) Audio call (BCIO:011022) Messaging (BCIO:011024) Email (BCIO:011025)
Online platform		Joy online platform available to support service users in finding local physical activity opportunities.	Website mode of delivery (BCIO:011027)

Behaviour Change Techniques (BCTs) characterised using the Behaviour Change Techniques Taxonomy v1 (BCTTv1). Modes of delivery characterised using the Mode of Delivery Ontology.

BCIO, Behaviour Change Intervention Ontology.

#### One-to-one consultation

All service users will receive one 45-minute one-to-one consultation with a PARS either in-person, via telephone call or online video call. This consultation will be guided by a physical activity, health and well-being questionnaire discussed in detail within the Outcome assessment section below. Consultations will feature Motivational Interviewing techniques: including Open questions, Affirming, Reflective listening and Summarising.^24^ These consultations will also identify and address barriers and facilitators to engaging in physical activity, structured by the COM-B model of behaviour change.^23^

#### Check-in contacts

Service users will receive three check-in contacts with their assigned PARS to check on progress and troubleshoot any issues taking part in physical activity. These check-in contacts will take place by email, phone or text.

#### Online platform

Service users will receive access to the Joy online platform (Pungo Ltd, London, UK) to find physical activity opportunities in their local area. The Joy online platform is a website interface that healthcare professionals (eg, GPs) can use to refer patients into health and social care services or members of the public can use to refer themselves to physical activity opportunities within their local area (https://www.thejoyapp.com/). PARS do not provide digital literacy and training on using the Joy online platform to service users, due to time and financial constraints.

### Physical activity referral specialists

Three PARS have been employed by Active in the Community CIC to deliver the Move Together Buckinghamshire programme over 3 years. The PARS will collaborate with local primary and secondary care services, local community organisations (eg, Bucks Mind, Leap) and Buckinghamshire Council to recruit Pathway service users to the programme. In the one-to-one consultation sessions, PARS will discuss perceived barriers and facilitators to physical activity with the service users and provide tailored recommendations for physical activity opportunities available within the local area.

PARS are required to hold the following specialist training qualifications: (i) Chartered Institute for the Management of Sport and Physical Activity Level 3 GP Exercise Referral qualification,^36^ (ii) Knowledge and training on the COM-B model of behaviour change,^23^ (iii) Making Every Contact Count training by Buckinghamshire Council,^37^ (iv) Mental Health First Aid training provided by Mind Buckinghamshire^38^ and (v) Active Medicine training.^39^ PARS are not required to hold any women’s health-specific training. However, all PARS will tailor all sessions and physical activity recommendations based on each individual service users’ pre-existing health conditions.

### Outcome assessments

An online questionnaire hosted by Active in the Community via Google Forms will be delivered to service users as part of the Move Together Buckinghamshire programme. This data will be analysed as secondary data for the programme evaluation. The questionnaire will be administered by the PARS to service users at baseline, during and at the end of the 12-week intervention period, and at 6 month and 12 month follow-ups. This questionnaire will be used as part of the intervention by the PARS to assess service users’ current physical activity, health and well-being. To maximise completion rates, service users will be offered the option to complete the questionnaire: (i) via verbal delivery by the PARS during consultations, where PARS will input service user data directly into the Google Form using a tablet or laptop, or (ii) by service users independently responding into the Google Form within or prior to the intervention session. Completion of the questionnaire should take 10–15 min of the 45-minute consultation sessions. This questionnaire has been pragmatically designed in collaboration with Buckinghamshire Council and service delivery partners to ensure all primary and secondary outcomes are accounted for, while minimising burden on service users and reducing time spent completing questionnaires during initial consultation and follow-up points. Translated versions of the questionnaire will be made available in the most common languages within the area: Urdu, Punjabi, Polish, Romanian, Portuguese and Tamil. The questionnaire is available on Open Science Framework: https://osf.io/2rt5a/files/gnjwr. In addition, demographic variables of age, gender and household postcode will be routinely collected via the Joy online platform and linked to service users using their individual study identification number.

### Primary outcome (physical activity)

The study’s primary outcome, physical activity, will be assessed with a ‘single item measure’ of physical activity,^40^ which asks: “In the past week, on how many days have you done a total of 30 minutes or more of physical activity, which was enough to raise your breathing rate? This may include sport, exercise, and brisk walking or cycling for recreation or to get to and from places but should not include housework or physical activity that may be part of your job”. Respondents can choose from 0 to 7 days. This measure has been found to have good test-retest reliability, has concurrent validity with other longer physical activity measures, is valid in comparison to accelerometer data^40–42^ and is appropriate for measuring shorter bouts of physical activity that may be accumulated throughout the day in older adults.^43^ This brief measure was chosen over longer alternatives, such as IPAQ-Short Form^44^ to facilitate completion within the time available during consultation sessions and to improve usability for service users and PARS.

A second question focusing on duration of moderate intensity activity will also be asked: “In the last 7 days, how much moderate intensity activity have you completed in total? Moderate activities refer to activities that take moderate physical effort and make you breathe somewhat harder than normal.” The question has four options: ‘Under 30 minutes’, ‘31-90 minutes’, ‘91-149 minutes’ and ‘150 minutes+’. Frequency of muscle-strengthening activities will be assessed with one item from the Muscle-Strengthening Exercise Questionnaire^45^ : “How many days, in a usual week, do you do muscle-strengthening exercise? eg, use weight machines, body weight exercises, resistance bands or free weights”. Respondents can choose from 0 to 7 days. This single item from the wider questionnaire was pragmatically chosen to provide data on muscle-strengthening activity recommended for older adults.^9^

### Secondary outcomes

#### Health-related quality of life

The EQ-5D-5L will be used to assess health-related quality of life across five dimensions: mobility, self-care, usual activities, pain/discomfort and anxiety/depression,^46^ with one question per domain. Responses will be answered using a 5-point Likert scale, ranging from ‘no problems’ to ‘inability to function’. Overall health will also be rated on a 0–100 visual analogue scale.^46^ Higher scores reflect greater quality of life across all items. Good convergent validity and reliability has been shown for the EQ-5D-5L in older adult populations.^47^

#### Loneliness

The Single-item Loneliness Scale will be used, which asks, “How often do you feel lonely?”.^48^ The question has five response options, ranging from ‘often or always’ to ‘never’. The measure has been shown to be valid as a brief screening tool for assessing loneliness in older adults.^49^

#### Confidence in engaging with physical activity

Four statements will be used to assess physical activity confidence,^50^ with each question having ten response options ranging from 1 (very low) to 10 (very high).

#### Use of healthcare services

Four questions will assess use of GP services, National Health Service 111/out-of-hours non-emergency services, Accident & Emergency and occurrence of trips or falls in the last 4 weeks. Each question has a ‘Yes’ or ‘No’ option. A 4-week recall period was selected for healthcare service use questions to minimise recall bias, as longer recall periods have been associated with reduced reporting accuracy among older adults.^51^

### Statistical analysis

Each service users’ questionnaire data will be linked across timepoints using unique individual study identification numbers. Descriptive statistics for outcomes will be reported across all timepoints. Outcome evaluation will be based on a within-subjects comparison between: (i) values at baseline and end of the 12-week intervention and (ii) values at the end of the 12-week intervention and 12-month follow-up. Sex-disaggregated analysis of outcomes will be undertaken as exploratory analysis. Linear mixed-effects regression models with time as a fixed effect, participant as a random effect, and age, gender and household postcode (or area-level deprivation) included as covariates will be used to estimate changes in physical activity, health-related quality of life, loneliness, physical activity confidence and use of healthcare services following introduction of Move Together Buckinghamshire.^52^ Data will be reported as means with 95% CIs, using a significance level of p<0.05. Data will be analysed using SPSS V.29 (IBM; Armonk, New York, USA).

### Process evaluation

A process evaluation will be undertaken to examine how the Move Together Buckinghamshire programme is implemented, focusing on whether it was delivered as intended and identifying factors that influenced its execution. The process evaluation will be completed in two phases: an initial assessment at 3 months into service delivery and an interim assessment at 15 months into service delivery. The process evaluation will explore the design, implementation, acceptability and contextual factors, which will contribute to decision-making about continued provision of the programme.

#### Focus groups

Two phases of participatory focus groups will be organised and conducted with service users 3 months into service delivery and 15 months into service delivery. Inclusion criteria for service users participating in the focus groups will be: (i) meeting the Move Together Buckinghamshire eligibility criteria specified above, (ii) taking part or have taken part in the Move Together Buckinghamshire programme and (iii) ability to converse in English.

In both phases, focus groups comprising 4–8 service users will take place either online or in-person in private, convenient locations such as local libraries and leisure centres. In-person focus groups will be audio-recorded using a smart tablet and online focus groups will be hosted and audio/video recorded via Microsoft Teams (Microsoft Corporation; Redmond, Washington, USA).

Focus groups will facilitate conversations between service users to identify: (i) how they have engaged with all aspects of the programme, including any barriers experienced, (ii) how acceptable they find each aspect of the programme and (iii) any issues requiring urgent improvement to the programme. All focus groups will last approximately 120 min and be delivered by two facilitators with previous experience facilitating focus groups in diverse populations. The facilitators will encourage conversation between service users, guided by a participatory focus group interview guide, including a range of participatory activities to promote engagement, build rapport with the group, foster group cohesion and mitigate hierarchical distinctions. Topics regarding acceptability have been developed in line with the Theoretical Framework of Acceptability (TFA) of healthcare interventions,^53^ exploring Affective Attitude, Burden, Ethicality, Intervention Coherence, Opportunity Costs, Perceived Effectiveness, Self-efficacy and General Acceptability. A £20 gift voucher will be supplied by Buckinghamshire Council to service users participating in focus groups.

#### Semi-structured interviews

Service users who cannot participate in the process evaluation focus groups due to travel or technology limitations will be offered an alternative semi-structured interview with a researcher. These interviews will take place via telephone, face-to-face at a location convenient to the service user, or online via Microsoft Teams. The semi-structured interview guide with service users will be in line with the focus group guide. A £20 gift voucher will be supplied by Buckinghamshire Council to service users participating in interviews.

Semi-structured interviews will also be conducted at 3 and 15 months into service delivery with PARS delivering the programme and strategic decision-makers who oversee the initiation, development and maintenance of Move Together Buckinghamshire. The interviews will explore: (i) perceptions of service user acceptability of all aspects of the programme structured using the TFA,^53^ (ii) what worked well for programme development and implementation and (iii) issues requiring improvement to the programme. A brief demographic characteristic questionnaire (age, sex, ethnicity, coaching experience) will be provided to PARS and strategic decision makers participating in the evaluation. Interviews will be conducted online via Microsoft Teams and last between 30 and 60 min.

### Data analysis

Qualitative data from the process evaluation will be analysed using NVivo V.14 (Lumivero; Denver, Colorado, USA) using the seven-phase Framework Method of Analysis.^54^ This analysis method involves (i) transcribing, (ii) re-reading the transcripts for familiarisation purposes, (iii) dual approaches to coding the transcripts using a combination of theoretically-led (ie, TFA^53^) and data-driven coding approaches (ie, inductively drawing on novel data produced), (iv) developing a working analytical framework, (v) applying the analytical framework, (vi) charting data into the framework matrix and (vii) interpreting the data.

### Economic analysis

An economic analysis will estimate the resources and costs required to design, set up and deliver the Move Together Buckinghamshire programme from a health system and participant perspective. Resource use will include staff time for programme design, recruitment of service users and PARS, materials, venue hire and delivery, as well as resources required for consultations and use of the Joy online platform. Out of pocket expenses borne by participants, including attending physical activity sessions, clothing and equipment, and travel, will also be collected as appropriate. The unit costs will be identified using administrative records, programme documentation and standard unit cost sources. Outcomes will include changes in physical activity and quality of life (EQ-5D-5L). Results will be presented using a cost-consequence framework,^55^ aligning costs with observed outcome changes at 12 weeks, 6 months and 12 months.

## Ethics and dissemination

A phased approach in response to project needs was adopted for obtaining ethical approval. Therefore, ethical approval was obtained from the College of Health, Medicine and Life Sciences Research Ethics Committee at Brunel University of London for the outcome evaluation (50864-LR-Jun/2025- 54 296–2) and process evaluation (51088-LR-Sep/2025-54622-1). All participants will receive information sheets and provide written informed consent to participate in this evaluation. All participants will be informed of their right to withdraw at any time. Anonymised raw data collected in this study will be uploaded to Brunel Figshare and Open Science Framework. The results of this study will be disseminated through peer-reviewed publications, scientific conferences, reports, webinars and local community outlets.

### Patient and public involvement

Patients or the public were not involved in the design, or conduct, or reporting or dissemination plans of our research.
