# Supplementary material for: Move Together Buckinghamshire: protocol for a pragmatic mixed-methods evaluation of a community physical activity programme for older adults with long-term conditions
Source: BMJ Open. 2026 Jul 31;16(7):e119845. doi: 10.1136/bmjopen-2026-119845 (PMC13436195; doi:10.1136/bmjopen-2026-119845)
Supplement: Supplementary file 1 [file bmjopen-2026-119845.reviewer_comments.pdf]

PEER REVIEW HISTORY

BMJ Open publishes all reviews undertaken for accepted manuscripts. Reviewers are asked to complete a checklist review form and are provided with free text boxes to elaborate on their assessment. These free text comments are reproduced below.

ARTICLE DETAILS

Title (Provisional)

Move Together Buckinghamshire: Protocol for a Pragmatic Mixed-Methods Evaluation of a Community Physical Activity Programme for Older Adults with Long-Term Conditions

Authors

Norris, Emma; Hings, Rebecca; Bailey, Daniel; Victor, Christina; Anokye, Nana; Banstola, Amrit; Androulaki Korakaki, Dynameni; Carrillo, Alejandra; Mansfield, Louise

---

VERSION 1 - REVIEW

**Reviewer**	**1**
**Name**	**Osman, Doaa A.**
**Affiliation**	**Department of Physical Therapy for Woman's Health, Cairo University, Physical Therapy for Woman's Health**
**Date**	**29-May-2026**
**COI**	**None**

The Move Together Buckinghamshire physical activity programme for adults over 50 years old with a long-term health condition living in deprived areas: A pragmatic pre-post study protocol

The protocol is strong, relevant, and well aligned with a high-impact journal, but it needs clearer reporting and tighter methodological justification. The main areas for improvement are sample size rationale, statistical methods, missing-data handling, selection bias, intervention fidelity, outcome measurement limits, and internal consistency across the manuscript.

The detailed comments below are intended to be constructive and to guide the authors toward a substantially strengthened revision:

1. **Title:**
    - Consider shortening the title while preserving essential information. I suggest

modifying the title to be: “Move Together Buckinghamshire: Protocol for a Pragmatic Mixed-Methods Evaluation of a Community Physical Activity Programme for Older Adults with Long-Term Conditions.”

2. **Abstract:**
    - The abstract does not mention the anticipated sample size or recruitment target, which limits readers’ ability to judge feasibility and statistical power. Please include the expected number of participants and rationale.
    - The abstract states that physical activity will be assessed at multiple timepoints but does not specify the measurement instrument used (the single-item physical activity measure).
3. **Strengths and limitations of this study:**
    - Although the absence of a control group is acknowledged, the justification provided is insufficient. The authors should explain more clearly why alternative comparator approaches, such as a waitlist control, matched comparator group, or comparator site, were not feasible within this pragmatic real-world evaluation.
    - A further limitation is that the evaluation does not include sex- or gender-disaggregated analyses of outcomes, nor does it systematically assess physical activity barriers related to common women’s health conditions (e.g., pelvic floor dysfunction, osteoporosis, post-mastectomy complications).
    - The English-language requirement for the qualitative process evaluation may exclude non-English-speaking participants, even though they can complete the questionnaire in translated formats. The authors should acknowledge this as a limitation because it may reduce the inclusivity and representativeness of the qualitative findings.
4. **Introduction:**
    - The opening statistic that physical activity prevents "at least 3.9 million premature deaths globally each year" cites Strain et al. (2020). Given the article was submitted in March 2026, more recent global burden estimates are available (e.g., the 2024 WHO Global Status Report on Physical Activity).
    - Given the likely relevance of sex- and gender-related factors to physical activity uptake and adherence in later life, the introduction would benefit from briefly acknowledging barriers commonly experienced by older women, particularly in relation to long-term condition management, confidence, and engagement with physical activity programmes.

- The theoretical basis of the intervention would benefit from clearer introduction-level contextualization. Briefly introducing the relevance of COM-B and Motivational Interviewing within the introduction would improve narrative flow and strengthen the coherence between the evidence base and programme design.
- The programme targets adults over 50 years, yet most physical activity guidelines reference distinct thresholds of 19–64 and 65+ years. Please justify the 50+ eligibility threshold in the introduction.

5. **Methods:**

- Design:
  - There appears to be a timeline inconsistency regarding follow-up assessments. The Design section refers to data collection at 6- and 12-month follow-ups (page 6), whereas the Outcome Assessments section later mentions 3-, 6-, and 12-month follow-ups (page 11). The authors should clarify and ensure consistency across the manuscript.
  - The manuscript does not specify adherence to a recognized reporting guideline for study protocols. The authors should clarify whether the protocol follows SPIRIT or another appropriate reporting framework relevant to the observational and qualitative components of the study.
- Service users:
  - Provide a formal sample size calculation based on effect sizes from comparable programmes (e.g., Active Herts, referenced as 21–24), or provide a clear rationale for why sample size estimation is not applicable, with reference to the pragmatic/service evaluation nature of the study.
  - Requiring participants to be "motivated to increase physical activity" may introduce self-selection bias because it preferentially recruits people who are already ready to change. This can limit representativeness and may inflate apparent feasibility or effectiveness compared with the broader target population.
  - The exclusion criteria include blood pressure >180/100 mmHg and resting heart rate >100 bpm. From a physical therapy and clinical safety perspective, it should be clarified:
    - o Who conducts this screening (PARS, referring GP, or self-report)?
    - o When are these measures taken (prior to referral, at initial consultation)?
    - o What happens if a service user presents with borderline values or if values change during the programme?

- Clarify whether women with pelvic floor dysfunction, urinary incontinence, prolapse, or recent pelvic surgery are included and, if so, whether tailored or modified physical activity advice will be provided.
- Additionally, conditions such as osteoporosis, fibromyalgia, osteoarthritis, and autoimmune disorders are highly prevalent among women over 50 years of age. Clarify whether individuals with these conditions are eligible for inclusion in the programme.

- Physical Activity Referral Specialists:
  - The protocol does not describe procedures for monitoring intervention fidelity across PARS staff. As variability in consultation delivery, motivational interviewing techniques, and follow-up contacts may influence outcomes, the authors should clarify whether any fidelity monitoring, supervision, standardisation procedures, or assessment tools will be used to ensure the programme is delivered consistently as intended.
  - Clarify whether PARS receive any women's health-specific training, particularly for conditions requiring modified exercise guidance in older women.
- Joy Online Platform:
  - The protocol does not adequately address digital literacy and accessibility considerations related to the Joy online platform, despite the target population comprising older adults living in deprived areas. Please clarify whether participants will receive support or orientation for platform use and whether engagement, non-use, or accessibility barriers will be monitored as part of the process evaluation.
- Table 1:
  - The table lists "Behavioural contract (BCT: 1.8)" for the online platform component. It is unclear how a behavioural contract is operationalised within the Joy platform. This should be explained either in the table or in the accompanying text.
  - "Adding objects to the environment (BCT: 12.5)" is listed for the online platform but this BCT typically refers to physical environmental modifications (e.g., placing exercise equipment in view). Its application to a digital platform requires explicit justification.
  - The consultation column lists "Audio call (BCIO:011022)" as a mode of delivery despite the consultation being described as a 45-minute in-person session elsewhere. This inconsistency should be resolved.

- Primary Outcome: Single-Item Physical Activity Measure:
  - The measure asks about days with "a total of 30 minutes or more" of physical activity. This 30-minute continuous threshold does not align with current UK Chief Medical Officers' guidance (referenced as [10]), which emphasizes that activity can be accumulated throughout the day. This could lead to systematic underestimation of physical activity in the target population, particularly older adults who may perform activity in shorter bouts.
  - Provide brief justification for not using alternative validated self-report measures commonly used in older adults (e.g., IPAQ-Short Form, PASE — Physical Activity Scale for the Elderly).

- Use of healthcare services:
  - The four-week recall period used to assess healthcare use may be too short to adequately capture service use in this population and could result in substantial floor effects. Justify the choice of this recall window or consider a longer assessment period, particularly at later follow-up timepoints.
  - Given the age and clinical profile of the target population, falls represent an important safety and functional outcome. The authors should either provide a stronger falls-related assessment approach or justify the use of the current brief self-report measure.
- Statistical Analysis:
  - The protocol does not clearly specify which baseline demographic variables will be collected. The authors should explicitly describe the demographic and clinical characteristics to be recorded at baseline, particularly variables relevant to participant profiling and statistical analyses.
  - Clarify the specific regression models to be used, the covariates to be included in the analyses, how clustering of participants within PARS will be addressed, and the planned approach for handling missing data.

6. References:

References 43 and 44 appear to cite the same paper. Remove duplication.

VERSION 1 - AUTHOR RESPONSE

Reviewer Comments	Response to Reviewer Comments
1. Title:<br>Consider shortening the title while preserving essential information. I suggest modifying the title to be: “Move Together Buckinghamshire:	Thank you for this suggestion. We have revised as you have suggested (Page 1).

Protocol for a Pragmatic Mixed-Methods Evaluation of a Community Physical Activity Programme for Older Adults with Long-Term Conditions."	
2. Abstract:<br>The abstract does not mention the anticipated sample size or recruitment target, which limits readers' ability to judge feasibility and statistical power. Please include the expected number of participants and rationale.	We have added clarification on this in the:<br>Abstract: "The programme's recruitment target is 3,000 service users over the 3-year programme." (page 1)<br>and main text: "The recruitment target set by Buckinghamshire Council is 3,000 service users over the three-year programme." (page 6)
The abstract states that physical activity will be assessed at multiple timepoints but does not specify the measurement instrument used (the single-item physical activity measure).	We have revised as you have suggested:<br>"A quantitative outcome evaluation will be conducted for secondary data routinely collected within the programme to assess the primary outcome of physical activity (using the single-item physical activity measure), and secondary outcomes of health-related quality of life, loneliness and confidence to engage in physical activity (assessed at baseline, during and at the end of the 12-week programme, and at 6-month and 12-month follow-up)." (Page 2).
3. Strengths and limitations of this study:<br>Although the absence of a control group is acknowledged, the justification provided is insufficient. The authors should explain more clearly why alternative comparator approaches, such as a waitlist control, matched comparator group, or comparator site, were not feasible within this pragmatic real-world evaluation.	We thank the reviewer for this comment and agree that the rationale for the absence of a comparator group should be made clearer. Buckinghamshire Council did not establish a control group, waitlist, matched comparator cohort, or comparator site during the design and implementation of the intervention. As a result, no suitable comparator data were available for the evaluation.<br><br>Alternative approaches such as a waitlist control were not feasible because the intervention was implemented as a service provision initiative rather than a research study, and access was not intentionally delayed for eligible participants. Similarly, matched comparator groups or comparator sites were not identified or established prospectively by the commissioning authority, and the data required to construct a robust retrospective comparator were unavailable.<br><br>We have revised the manuscript to clarify these constraints: "A comparator group was not included as no such group was established by Buckinghamshire Council during intervention implementation." (Page 6).
A further limitation is that the evaluation does not include sex- or gender-disaggregated	We have added that:<br>"Sex-disaggregated analysis of outcomes will be

analyses of outcomes, nor does it systematically assess physical activity barriers related to common women's health conditions (e.g., pelvic floor dysfunction, osteoporosis, post-mastectomy complications).	presented." (page 14)<br><br>We have added that this study does not explore health barriers related to common women's health conditions within the study limitations: "This study does not assess physical activity barriers related to common women's health conditions." (page 3)
The English-language requirement for the qualitative process evaluation may exclude non-English-speaking participants, even though they can complete the questionnaire in translated formats. The authors should acknowledge this as a limitation because it may reduce the inclusivity and representativeness of the qualitative findings.	We have included this within the limitations section: "This study also includes English-speaking participants only, although translated questionnaires were provided." (page 3)
4. Introduction:<br>The opening statistic that physical activity prevents "at least 3.9 million premature deaths globally each year" cites Strain et al. (2020). Given the article was submitted in March 2026, more recent global burden estimates are available (e.g., the 2024 WHO Global Status Report on Physical Activity).	Thanks for this comment. To our knowledge, this is the most up-to-date global estimate of physical activity-related mortality reduction. The 2024 WHO Global Status Report on Physical Activity does not contain estimates related to mortality. The more recent physical inactivity estimate paper by Strain et al. 2024 (Lancet Global Health) also does not have mortality data. We have hence maintained the 2020 Strain paper.
Given the likely relevance of sex- and gender-related factors to physical activity uptake and adherence in later life, the introduction would benefit from briefly acknowledging barriers commonly experienced by older women, particularly in relation to long-term condition management, confidence, and engagement with physical activity programmes.	We have added reference to this:<br>"These socioeconomic inequalities may be compounded by sex-related differences in physical activity, with older women typically reporting lower activity levels and experiencing distinct barriers to participation compared with older men (14)." (page 4)
The theoretical basis of the intervention would benefit from clearer introduction-level contextualization. Briefly introducing the relevance of COM-B and Motivational Interviewing within the introduction would improve narrative flow and strengthen the coherence between the evidence base and programme design.	We have added discussion on this within the introduction:<br>"Additionally, behaviour change interventions informed by theory may be particularly effective for increasing physical activity in older adults. The Capability, Opportunity, Motivation–Behaviour (COM-B) model provides a framework for understanding barriers and facilitators to physical activity (Meredith et al. 2023; Michie et al. 2011), while Motivational Interviewing offers a person-centred approach to enhancing motivation and supporting sustained behaviour change (Zhu et al. 2024)." (page 5)
The programme targets adults over 50 years, yet most physical activity guidelines reference distinct thresholds of 19–64 and 65+ years.	We thank the reviewer for this comment. The age range was determined by Buckinghamshire Council as the intervention developer.

Please justify the 50+ eligibility threshold in the introduction.	We have added clarity on this age group in the introduction:<br>"Interventions are, therefore, needed to increase physical activity from midlife and into older adulthood to reduce the associated high burden of long-term conditions (Centre for Ageing Better, 2021)." (page 4)
5. Methods:<br>Design: There appears to be a timeline inconsistency regarding follow-up assessments. The Design section refers to data collection at 6- and 12-month follow-ups (page 6), whereas the Outcome Assessments section later mentions 3-, 6-, and 12-month follow-ups (page 11). The authors should clarify and ensure consistency across the manuscript.	Thank you for this comment. We have revised to more accurately reflect data collection at 6- and 12-month follow-up for quantitative data, as per Figure 1: "The questionnaire will be administered by the PARS to service users at baseline, during and at the end of the 12-week intervention period, and at 6-month and 12-month follow-ups." (Page 11)
The manuscript does not specify adherence to a recognized reporting guideline for study protocols. The authors should clarify whether the protocol follows SPIRIT or another appropriate reporting framework relevant to the observational and qualitative components of the study.	This is a pragmatic, non-randomised study and hence the SPIRIT protocol guidance for randomised interventions is not relevant here. No other reporting guidelines on the EQUATOR network are appropriate for this study design.
Service users:<br>Provide a formal sample size calculation based on effect sizes from comparable programmes (e.g., Active Herts, referenced as 21–24), or provide a clear rationale for why sample size estimation is not applicable, with reference to the pragmatic/service evaluation nature of the study.	We have added this clarification: "Sample size calculations were not performed due to the pragmatic and service evaluation nature of this study." (Page 6)
Requiring participants to be "motivated to increase physical activity" may introduce self-selection bias because it preferentially recruits people who are already ready to change. This can limit representativeness and may inflate apparent feasibility or effectiveness compared with the broader target population.	Thanks for this comment. Motivation is a key determinant of behaviour change, as depicted by the COM-B model. Buckinghamshire Council included motivation to be more active as an inclusion criteria to maximise the likelihood of service users engaging with the service. We have added this as a limitation:<br>"This study also recruits service users that are motivated to increase their physical activity which may limit representativeness to the wider population." (page 3)
- The exclusion criteria include blood pressure >180/100 mmHg and resting heart rate >100 bpm. From a physical therapy and clinical safety perspective, it should be clarified:<br>o Who conducts this screening (PARS, referring GP, or self-report)?<br>o When are these measures taken (prior to referral, at initial consultation)?	Thank you for this important comment. We have added a new paragraph to address the different referral routes and how inclusion criteria is assessed:<br>"The process of screening service users for inclusion criteria differ will depending on the route of referral into the programme. If a health professional (e.g., GP) conducts the

o What happens if a service user presents with borderline values or if values change during the programme?	screening, they are agreeing that the individual's condition is controlled and they are suitable to take part. When a person self-refers or is referred from a non-clinical setting (e.g., an exercise class, a local community centre), the Physical Activity Referral Specialist (PARS) working with the service user will conduct a screening during their initial consultation. or will redirect the service user to their GP to assess their suitability in borderline cases. PARS monitor any changes in service users' long-term health conditions throughout the programme and take appropriate action where such changes affect eligibility to continue participation." (page 7)
Clarify whether women with pelvic floor dysfunction, urinary incontinence, prolapse, or recent pelvic surgery are included and, if so, whether tailored or modified physical activity advice will be provided.	Women with pelvic floor dysfunction, urinary incontinence, prolapse, or recent pelvic surgery are not included in the list of long-term health conditions currently. We have added clarity on this in the paper:<br>"Women with pelvic floor dysfunction, urinary incontinence, prolapse or recent pelvic surgery are not included in the list of long-term health conditions inclusion criteria." (page 7)
Additionally, conditions such as osteoporosis, fibromyalgia, osteoarthritis, and autoimmune disorders are highly prevalent among women over 50 years of age. Clarify whether individuals with these conditions are eligible for inclusion in the programme.	Thank you for this comment. People with osteoporosis, osteoarthritis, and fibromyalgia (classified under musculoskeletal conditions) are eligible for inclusion in the programme. Although "autoimmune disorders" is not listed for inclusion, Multiple Sclerosis is listed as a long-term health condition eligible for inclusion.<br><br>"ii) living with a long-term health condition (such as cancer, dementia, diabetes [controlled], hypertension, multiple sclerosis, stroke and musculoskeletal conditions such as osteoporosis, osteoarthritis and fibromyalgia)," (page 7)
Physical Activity Referral Specialists:<br>The protocol does not describe procedures for monitoring intervention fidelity across PARS staff. As variability in consultation delivery, motivational interviewing techniques, and follow-up contacts may influence outcomes, the authors should clarify whether any fidelity monitoring, supervision, standardisation procedures, or assessment tools will be used to ensure the programme is delivered consistently as intended.	We have clarified that no fidelity assessment is being performed within this evaluation:<br>"No fidelity assessment will be performed within this evaluation." (page 8)

Clarify whether PARS receive any women's health-specific training, particularly for conditions requiring modified exercise guidance in older women.	The PARS are not required to complete women's health-specific training as part of the Move Together Buckinghamshire programme. We have added clarification on this:<br>"PARS are not required to hold any women's health-specific training. However, all PARS will tailor all sessions and physical activity recommendations based on each individual service users' pre-existing health conditions." (page 9)
Joy Online Platform:<br>The protocol does not adequately address digital literacy and accessibility considerations related to the Joy online platform, despite the target population comprising older adults living in deprived areas. Please clarify whether participants will receive support or orientation for platform use and whether engagement, non-use, or accessibility barriers will be monitored as part of the process evaluation.	The service delivery team do not provide digital literacy and accessibility training for how to use the Joy online platform to service users due to time and financial constraints.<br>"PARS do not provide digital literacy and training on using the Joy online platform to service users due to time and financial constraints." (page 8)<br><br>We have also added clarity within the process evaluation section that all aspects of the programme will be explored within focus groups and interviews:<br>"Focus groups will facilitate conversations between service users to identify: i) how they have engaged with all aspects of the programme, including any barriers experienced, ii) how acceptable they find each aspect of the programme, and iii) any issues requiring urgent improvement to the programme." (page 15)<br><br>"The interviews will explore: i) perceptions of service user acceptability of all aspects of the programme structured using the Theoretical Framework of Acceptability (48), ii) what worked well for programme development and implementation, and iii) issues requiring improvement to the programme." (page 15)
Table 1:<br>The table lists "Behavioural contract (BCT: 1.8)" for the online platform component. It is unclear how a behavioural contract is operationalised within the Joy platform. This should be explained either in the table or in the accompanying text.	We have removed this from the table.
"Adding objects to the environment (BCT: 12.5)" is listed for the online platform but this BCT typically refers to physical environmental modifications (e.g., placing exercise equipment in view). Its application to a digital platform	We have removed this from the table.

requires explicit justification.	
The consultation column lists "Audio call (BCIO:011022)" as a mode of delivery despite the consultation being described as a 45-minute in-person session elsewhere. This inconsistency should be resolved.	Thank you for highlighting this discrepancy. This has been resolved with the following text: "All service users will receive one 45-minute one-to-one consultation with a PARS either in-person, via telephone call, or online video call." (page 8)
Primary Outcome: Single-Item Physical Activity Measure:<br>The measure asks about days with "a total of 30 minutes or more" of physical activity. This 30-minute continuous threshold does not align with current UK Chief Medical Officers' guidance (referenced as [10]), which emphasizes that activity can be accumulated throughout the day. This could lead to systematic underestimation of physical activity in the target population, particularly older adults who may perform activity in shorter bouts.	Thanks for this comment. The 30-minute in the item refers to accumulation across the day, including shorter bouts of activity, making it appropriate to measure older adults' activity. Validation studies have shown the single-item measure to be a valid, pragmatic tool for assessing older adults' physical activity (Macdonald et al. 2021).<br><br>We have added clarification on this into the manuscript:<br>"This measure has been found to have good test-retest reliability, has concurrent validity with other longer physical activity measures, is valid in comparison to accelerometer data (40–42) and is appropriate for measuring shorter bours of physical activity that may be accumulated throughout the day in older adults (43). (page 12)
Provide brief justification for not using alternative validated self-report measures commonly used in older adults (e.g., IPAQ-Short Form, PASE — Physical Activity Scale for the Elderly).	Thanks for this comment. We have added clarification on this in the paper:<br>"This brief measure was chosen over longer alternatives (e.g., IPAQ-Short Form (Craig et al. 2003) to facilitate completion within the time available during consultation sessions and to improve usability for service users and PARS."<br>(page 12)
Use of healthcare services:<br>The four-week recall period used to assess healthcare use may be too short to adequately capture service use in this population and could result in substantial floor effects. Justify the choice of this recall window or consider a longer assessment period, particularly at later follow-up timepoints.	We thank the reviewer for this comment. The four-week recall period was selected to minimise recall bias and improve the accuracy of self-reported healthcare utilisation, particularly given the age of the study population and the range of outcomes being assessed. While we acknowledge that a longer recall period may capture a greater number of healthcare contacts, longer recall periods are also associated with reduced reporting accuracy of falls (Cummings et al. 1998). We have added reference to this in the paper:<br>"A four-week recall period was selected for healthcare service use questions to minimise recall bias, as longer recall periods have been associated with reduced reporting accuracy among older adults (Cummings et al., 1988)."<br>(page 13)

Given the age and clinical profile of the target population, falls represent an important safety and functional outcome. The authors should either provide a stronger falls-related assessment approach or justify the use of the current brief self-report measure.	The questionnaire includes one question on occurrence of trips or falls in the last four weeks (page 13).<br><br>This approach was agreed in collaboration with Buckinghamshire Council and service delivery partners to ensure all primary and secondary outcomes are accounted for whilst minimising burden on service users and reducing time spent completing questionnaires during initial consultation and follow-up points.<br><br>We have added discussion of this in-text:<br>"This questionnaire has been pragmatically designed in collaboration with Buckinghamshire Council and service delivery partners to ensure all primary and secondary outcomes are accounted for, whilst minimising burden on service users and reducing time spent completing questionnaires during initial consultation and follow-up points." (page 12)
Statistical Analysis:<br>The protocol does not clearly specify which baseline demographic variables will be collected. The authors should explicitly describe the demographic and clinical characteristics to be recorded at baseline, particularly variables relevant to participant profiling and statistical analyses.	Age, gender and postcode to determine area of residency within Buckinghamshire are the only demographic variables routinely collected, done via the Joy online platform. The rationale for this is that demographic variables could not be collected and analysed within secondary data analysis procedures and Brunel ethical approvals.<br><br>We have added clarity on this within the manuscript:<br>"In addition, demographic variables of age, gender and household postcode will be routinely collected via the Joy online platform and linked to service users using their individual study identification number." (page 11)
Clarify the specific regression models to be used, the covariates to be included in the analyses, how clustering of participants within PARS will be addressed, and the planned approach for handling missing data.	This has been clarified in the paper:<br>"Linear mixed-effects regression models with time as a fixed effect, participant as a random effect, and age, gender and household postcode (or area-level deprivation) included as covariates will be used to estimate changes in physical activity, health-related quality of life, loneliness, physical activity confidence and use of healthcare services following introduction of Move Together Buckinghamshire (47)." (page 14)
6. References:<br>• References 43 and 44 appear to cite the same	We have removed this duplication.

paper. Remove duplication.	
